# U shaped relationship between serum glucose potassium ratio and mortality in critically ill patients with toxic encephalopathy

**DOI:** 10.1038/s41598-025-12496-4

**Published:** 2025-07-23

**Authors:** Lei Sun, Feng Shao, Ting Liu, Ping Jin

**Affiliations:** Department of Critical Care Medicine, Jingzhou Hospital Affiliated to Yangtze University, No.26 Chuyuan Avenue, Jingzhou District, Jingzhou, 434020 Hubei Province China

**Keywords:** Toxic encephalopathy, Glucose-potassium ratio, Clinical outcomes, Mortality, Neurology, Neurological disorders

## Abstract

The link between serum glucose-potassium ratio (GPR) and mortality in critically ill toxic encephalopathy (TE) patients is not well defined. This study has aimed to clarify the effect of GPR fluctuations on TE mortality. A total of 3,462 TE patients with TE requiring ICU care were selected from the Medical Information Mart Intensive Care (MIMIC-IV) database. Patients were categorized into three groups based on GPR tertiles: Tertile 1 (*n* = 1154, range 0.103–1.442), Tertile 2 (*n* = 1153, range 1.444–1.966), and Tertile 3 (*n* = 1155, range 1.967–12.937). The primary outcomes studied were 28-day and 90-day all-cause mortality (ACM). To analyze the relationship between GPR and outcomes, we employed Cox regression models adjusted for multiple covariates and restricted cubic splines to explore the potential non-linear association. The 3,462 - patient cohort had a mean age of 67.7 ± 16.6 years, with 58.2% male. The 28-day and 90-day ACM were 21.9% and 31.2%, respectively. Multivariate adjusted analysis showed no overall GPR–ACM correlation at 28 and 90 d. Regarding different groups, with T2 as the reference group (Ref), for 28-day ACM, the adjusted hazard ratio (HR) of the T1 was 1.20 (95% Confidence Interval [CI]: 1.00–1.44, p ≡ *P* = 0.049), and that of T3 group was 1.22 (95% CI: 1.01–1.47, *P* = 0.035). For 90 - day ACM, the adjusted HR of the T1 was 1.19 (95% CI: 1.02–1.39, *P* = 0.023), and the T3 was 1.20 (95% CI: 1.03–1.40). The correlation between the GPR lesvel and ACM was U-shaped association. The left and right - hand side effect sizes at the inflection point (1.65) were 0.472 (HR: 0.472, 95% CI 0.306–0.728, *P* < 0.001) and 1.127 (HR: 1.127, 95% CI 1.032–1.229, *P* = 0.0075). Sensitivity analysis was stable. Our findings have revealed a U-shaped relationship between GPR levels and ACM in critically ill patients with TE. Close attention should therefore be paid to this issue in order to improve patient care.

**Supplementary Information:**

The online version contains supplementary material available at 10.1038/s41598-025-12496-4.

## Introduction

Toxic encephalopathy (TE) is a type of central nervous system dysfunction caused by various neurotoxins, such as heavy metals, organic solvents, and other chemical substances^1–4^. Acute TE often presents with symptoms, such as headache, dizziness, and somnolence. In severe cases, unconsciousness, convulsions, coma, or even death^5^. Its prognosis is affected by multiple factors, including the severity of poisoning, exposure time, timeliness of treatment, and individual physiological and genetic differences^6^. Although current treatment strategies have improved patient prognosis to a certain extent, high rates of disability and mortality remain. In addition, the clinical manifestations of TE are diverse and also lack specificity, posing challenges for early diagnosis and prognosis assessment^6,7^.

The glucose–potassium ratio (GPR) has emerged as a potential biomarker in recent years, and its alterations are associated with various pathological processes, particularly acute brain injury, metabolic disorders, and inflammatory responses^8–10^. Elevated GPR levels are implicated in cellular metabolic dysfunction and stress responses. For instance, in studies on Delayed Neuropsychiatric Syndrome (DNS) following carbon monoxide (CO) poisoning, GPR has been identified as a potential predictor of DNS development, with higher GPR levels correlating significantly with disease severity^11,12^. Recent studies have shown that higher GPR is associated with increased mortality at 28 and 90 days in patients with acute ischemic stroke (AIS)^13^. In addition, GPR has been shown to be associated with hemorrhagic transformation and stroke recurrence in patients with ischemic stroke^14^.

Given the critical roles of GPR in cellular metabolism and stress responses^15^, and considering that the pathophysiology of TE involves metabolic disturbances and oxidative stress^6^, a potential correlation between GPR and the prognosis warrants investigation. Elevated GPR levels may indicate impaired cellular metabolism, which can affect neuronal repair and functional recovery. In contrast, changes in GPR may reflect the intensity of inflammatory responses, which play a key role in the progression of toxic encephalopathy. Therefore, exploring the correlation between GPR and the prognosis of toxic encephalopathy could not only facilitate the early identification of high-risk patients, but also provide a basis for personalized treatment strategies. This study has primarily investigated the correlation between the GPR and mortality rates among critically ill patients with TE.

## Materials and methods

### Data sources

This study has utilized data sourced from the Medical Information Market for Intensive Care IV (MIMIC-IV) database version 3.1^16^. This is an extensive, publicly accessible repository that archives comprehensive clinical data from more than 200,000 intensive care unit (ICU) admissions at the Beth Israel Deaconess Medical Center in Boston, Massachusetts, from 2008 to 2022. The database includes a broad spectrum of patient information such as demographic details, vital signs, laboratory test outcomes, treatment procedures, and clinical documentation, all gathered as part of standard patient care. The MIMIC-IV database was approved by the review boards of the Massachusetts Institute of Technology and Beth Israel Deaconess Medical Center, with all of the sensitive health data anonymized to safeguard patient confidentiality. Access to the database for this research was authorized by Lei Sun (authorization number: 57321424). The findings of this study are in accordance with the guidelines established by the Strengthening the Reporting of Observational Studies in Epidemiology (STROBE)^17^.

### Study population

**Fig. 1:**
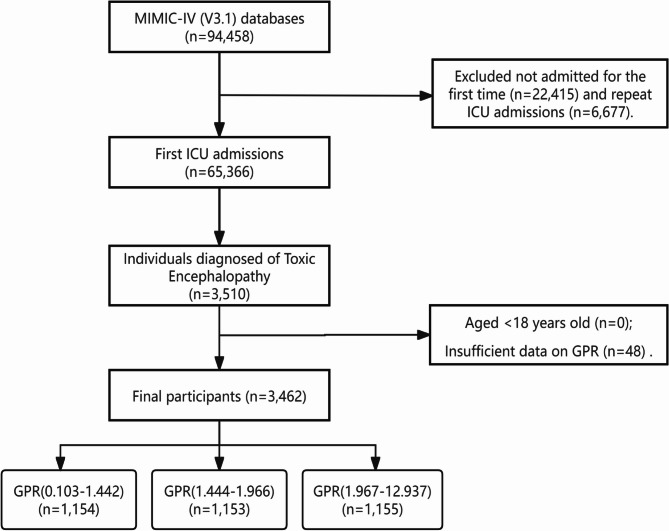
Flow diagram of the sample selection from the Medical Information Mart for Intensive Care IV (MIMIC-IV) database (version 3.1).

We focused on adults (18 years and older) diagnosed with toxic encephalopathy (TE) as to the International Classification of Diseases, 9th and 10th editions (ICD-9 and ICD-10)^18^, specifically those with ICD-9 code 349.82 and ICD-10 codes G92, G92.8, and G92.9. Initially, we identified 3,510 TE patients who had their initial intensive care unit (ICU) admission. We excluded patients under 18 years of age and those for whom data necessary for calculating the glucose-potassium ratio (GPR) were missing. After applying the exclusion criteria, the study cohort comprised of 3,462 eligible patients (Fig. 1).

### Data extraction

We specified that the initial laboratory results recorded within the first 24 h of ICU admission were used for performed analysis. Ultimately, we collected the following data: (1) Demographic information, including age and sex; (2) Physiological parameters, such as heart rate, mean arterial pressure (MAP), respiratory rate (RR), and oxygen saturation (SpO₂); (3) Clinical severity indices, including the Glasgow Coma Scale (GCS), Sequential Organ Failure Assessment (SOFA), Simplified Acute Physiological Score (SAPS-II), and Oxford Acute Severity of Illness Score (OASIS); (4) Hematological and biochemical markers, including hemoglobin (Hb), red blood cell count (RBC), white blood cell count (WBC), platelet count (PLT), activated partial thromboplastin time (APTT), glucose, blood urea nitrogen (BUN), serum creatinine (SCr), potassium (K), and sodium (Na); and (5) Pre-existing comorbidities, such as heart failure (HF), peripheral vascular disease (PVD), cardiovascular disease (CVD), renal disease, hepatic disorders, and diabetes mellitus (DM).

### Clinical outcomes

The main objective of this study was to evaluate the 28-day and 90-day all-cause mortality (ACM). Importantly, the determination of mortality was based on whether patients passed away within a specified timeframe after being admitted to the intensive care unit (ICU) and not just on observing a reduction in the patient numbers at a particular time.

### Statistical analyses

Continuous data were presented using the mean ± standard deviation (SD) or median and interquartile range (IQR), while categorical data were expressed in terms of counts or percentages. To compare baseline characteristics, continuous data were analyzed using the Mann–Whitney *U* test, and categorical data were compared using the chi-square test. Patients in this study were categorized into three groups based on GPR tertiles: Tertile 1 (*n* = 1154, range 0.103–1.442), Tertile 2 (*n* = 1153, range 1.444–1.966), and Tertile 3 (*n* = 1155, range 1.967–12.937). To investigate the correlation between GPR and post-ICU mortality, smooth curve fitting was performed along with both unadjusted and adjusted multivariate Cox regression analyses in order to assess the robustness of the relationship. Hazard ratios (HR) and 95% confidence intervals (CIs) were computed to quantify the strength of associations. Variables were considered for adjustment if they altered the hazard ratios by at least 10% based on clinical considerations^19^. We utilized the variance inflation factor (VIF) method in order to evaluate multicollinearity, considering a VIF value of five or higher as indicative of multicollinearity. Our analysis confirmed that none of the variables exhibited multicollinearity (Supplementary Table S1). The results were initially adjusted for age and sex in Model I. Model II included further adjustments for mean arterial pressure (MAP), respiratory rate (RR), oxygen saturation (SpO₂), and Glasgow Coma Scale (GCS). Model III included additional adjustments for heart failure (HF); peripheral vascular disease (PVD) cardiovascular disease (CVD); renal disease; hepatic disorders; and diabetes mellitus (DM). Model IV involved the most extensive adjustments for blood urea nitrogen (BUN), hemoglobin (Hb), sodium (Na), red blood cell count (RBC), platelet count (PLT), activated partial thromboplastin time (APTT), and serum creatinine (SCr). Threshold effect analyses were performed to evaluate the predictive power of GPR for ACM.

All comparisons were pre-specified, and two-sided tests were used, with a significance level set at *P* < 0.05, to indicate statistical significance between two or more groups. Covariates (continuous variables with means) were used to interpolate the missing values (Supplementary Table S2). All of the performed analyses were conducted using R version 3.3.2 (available at http://www.R-project.org, R Foundation) and Free Statistics version 2.1.

## Results

### Baseline characteristics of patients

Table 1 shows the significant associations between GPR and various clinical characteristics, comorbidities, disease severity, and outcomes. The mean age was 67.7 ± 16.6 years old, of whom 1932 (55.8%) were male. Higher GPR levels were associated with older age. MAP and SpO₂ did not show significant differences across the groups (MAP, *P* = 0.085; SpO₂: *P* = 0.236). Other laboratory parameters, including Hb, RBC, WBC, PLT, APTT, and Na levels showed some differences across the groups, but with less pronounced trends. The prevalence of HF, CVD, renal disease, and DM was higher in T3 (HF, 35.5%; CVD, 22%; renal disease, 30.2%; DM, 58.4%) than in the T1 and T2 groups (HF, 29.6%; CVD, 14.2%; renal disease, 23.8%; DM, 21.8% and 26.7%, respectively; *P* < 0.001 for all). Higher GPR levels were associated with increased disease severity, as evidenced by lower GCS scores (T3:10.2 vs. T1:10.9 and T2:10.7, *P* < 0.001) and higher SOFA, SAPS II, and OASIS scores at T3. The 28-day and 90-day ACM were 21.9% and 31.2%, respectively.

**Table 1:** Baseline characteristics of the study population.

Variables	GPR	T1 (0.103–1.442)	T2 (1.444–1.966)	T3 (1.967–12.937)	*P*-value
	Total (*n* = 3462)	(*n* = 1154)	(*n* = 1153)	(*n* = 1155)	
GPR	1.9 ± 0.9	1.2 ± 0.2	1.7 ± 0.1	2.7 ± 1.0	< 0.001
Age (year)	67.7 ± 16.6	66.0 ± 18.4	67.7 ± 16.8	69.3 ± 14.3	< 0.001
Male (n,%)	1932 (55.8)	672 (58.2)	646 (56)	614 (53.2)	0.048
**Vital signs**					
Heart rate (beats/min)	87.5 ± 16.9	86.4 ± 16.7	87.4 ± 17.0	88.8 ± 16.9	0.002
MAP (mmHg)	79.9 ± 11.3	79.4 ± 11.5	80.4 ± 11.2	79.8 ± 11.2	0.085
RR (breath/min)	20.2 ± 4.2	19.9 ± 4.2	20.1 ± 4.1	20.7 ± 4.3	< 0.001
SpO2 (%)	96.7 ± 2.4	96.7 ± 2.6	96.8 ± 2.1	96.6 ± 2.4	0.236
**Laboratory parameters**					
Hb (g/dL)	10.2 ± 2.1	10.1 ± 2.0	10.4 ± 2.1	10.1 ± 2.0	0.005
RBC (10^9^/L)	3.4 ± 0.7	3.4 ± 0.7	3.5 ± 0.7	3.4 ± 0.7	0.031
WBC (10^9^/L)	10.7 (7.6, 14.8)	9.9 (7.1, 13.9)	10.6 (7.6, 14.5)	11.4 (8.2, 15.9)	< 0.001
PLT (10^9^/L)	189.4 ± 105.1	188.1 ± 100.2	186.0 ± 100.5	194.0 ± 113.8	0.163
APTT (sec)	38.4 ± 20.5	38.7 ± 20.3	37.7 ± 19.3	38.7 ± 21.9	0.42
Na (mmol/L)	139.3 ± 6.0	139.0 ± 5.8	139.3 ± 5.6	139.5 ± 6.6	0.087
Glucose (mmol/L)	7.6 ± 3.3	5.2 ± 1.0	6.8 ± 1.0	10.8 ± 3.7	< 0.001
K (mmol/L)	4.1 ± 0.6	4.4 ± 0.7	4.1 ± 0.5	4.0 ± 0.6	< 0.001
BUN (mg/dL)	24.0 (14.0, 42.0)	24.0 (13.0, 44.0)	22.0 (13.0, 35.0)	28.0 (17.0, 45.0)	< 0.001
SCr (mg/dL)	1.3 (0.9, 2.3)	1.4 (0.9, 2.6)	1.2 (0.8, 2.0)	1.4 (0.9, 2.4)	< 0.001
**Comorbidities**					
HF (n, %)	1087 (31.4)	342 (29.6)	335 (29.1)	410 (35.5)	0.001
PVD (n, %)	350 (10.1)	118 (10.2)	113 (9.8)	119 (10.3)	0.911
CVD (n, %)	633 (18.3)	164 (14.2)	215 (18.6)	254 (22)	< 0.001
Renal disease (n, %)	958 (27.7)	335 (29)	274 (23.8)	349 (30.2)	0.001
Hepatic disorders (n, %)	604 (17.4)	197 (17.1)	197 (17.1)	210 (18.2)	0.722
DM (n, %)	1233 (35.6)	251 (21.8)	308 (26.7)	674 (58.4)	< 0.001
**Clinical severity**					
GCS	10.6 ± 3.9	10.9 ± 3.8	10.7 ± 3.8	10.2 ± 3.9	< 0.001
SOFA	6.0 ± 3.6	6.0 ± 3.7	5.7 ± 3.4	6.3 ± 3.6	< 0.001
SAPSII	42.3 ± 14.6	42.2 ± 15.9	40.7 ± 13.7	43.9 ± 14.1	< 0.001
OASIS	36.7 ± 9.3	36.1 ± 9.1	36.4 ± 8.9	37.6 ± 9.7	< 0.001
**Outcomes**					
28d-ACM (n, %)	759 (21.9)	265 (23)	215 (18.6)	279 (24.2)	0.003
90d-ACM (n, %)	1081 (31.2)	370 (32.1)	314 (27.2)	397 (34.4)	< 0.001

GPR, glucose potassium ratio; MAP, mean arterial pressure; RR, respiratory rate; SpO₂, oxygen saturation; Hb, hemoglobin; RBC, red blood cell count; WBC, white blood cell count; PLT, platelet count; APTT, activated partial thromboplastin time; BUN, blood urea nitrogen; SCr, serum creatinine; K, potassium; Na, sodium; HF, heart failure; PVD, peripheral vascular disease; CVD, cardiovascular disease; DM, diabetes mellitus; GCS, Glasgow Coma Scale; SOFA, Sequential Organ Failure Assessment; SAPS- II, Simplified Acute Physiological Score- II; OASIS, Oxford Acute Severity of Illness Score; 28d-ACM, 28-day all-cause mortality; 90d-ACM, 90-day all-cause mortality.

### Association between GPR and outcome

In the multivariate Cox regression analysis (Table 2), the relationship between GPR levels and mortality was found to be not significant when GPR was treated as a continuous variable for either the 28-day or 90-day ACM. However, when the GPR was categorized and the T2 group was used as the reference category, significant differences in mortality were observed across the GPR groups. For 28-day ACM, the HR for the T1 group was 1.28 (95% CI: 1.07–1.53, *P* = 0.008), and for the T3 group, it was 1.35 (95% CI: 1.13–1.61, *P* = 0.001). After adjustment, the HR for the T1 group was 1.20 (95% CI: 1.00–1.44, *P* = 0.049) and for the T3 group was 1.22 (95% CI: 1.01–1.47, *P* = 0.035). For 90-day ACM, the unadjusted HR for the T1 group was 1.23 (95% CI: 1.06–1.43, *P* = 0.007), and for the T3 group, it was 1.34 (95% CI: 1.15–1.55). After adjustment, the HR for the T1 group was 1.19 (95% CI: 1.02–1.39, *P* = 0.023), and for the T3 group, it was 1.20 (95% CI: 1.03–1.40).

**Table 2:** Association between GPR and mortality.

Variable	Crude		Modle I		Modle II		Modle III		Modle IV	
	HR (95%CI)	*P*-value	HR (95%CI)	*P*-value	HR (95%CI)	*P*-value	HR (95%CI)	*P*-value	HR (95%CI)	*P*-value
**28d-ACM**										
GPR	1.04 (0.96 ~ 1.13)	0.368	1.03 (0.95 ~ 1.12)	0.499	1 (0.92 ~ 1.08)	0.945	1.03 (0.95 ~ 1.12)	0.495	1.02 (0.94 ~ 1.11)	0.567
Tertile										
T1	1.28 (1.07 ~ 1.53)	0.008	1.32 (1.1 ~ 1.58)	0.003	1.32 (1.1 ~ 1.58)	0.003	1.27 (1.06 ~ 1.53)	0.009	1.2 (1 ~ 1.44)	0.049
T2	1(Ref)		1(Ref)		1(Ref)		1(Ref)		1(Ref)	
T3	1.35 (1.13 ~ 1.61)	0.001	1.32 (1.11 ~ 1.58)	0.002	1.24 (1.04 ~ 1.48)	0.018	1.3 (1.08 ~ 1.57)	0.005	1.22 (1.01 ~ 1.47)	0.035
**90d-ACM**										
GPR	1.05 (0.98 ~ 1.12)	0.195	1.04 (0.97 ~ 1.11)	0.277	1.02 (0.95 ~ 1.09)	0.662	1.03 (0.96 ~ 1.1)	0.448	1.02 (0.95 ~ 1.1)	0.561
Tertile										
T1	1.23 (1.06 ~ 1.43)	0.007	1.28 (1.1 ~ 1.48)	0.001	1.27 (1.1 ~ 1.48)	0.002	1.25 (1.08 ~ 1.46)	0.004	1.19 (1.02 ~ 1.39)	0.023
T2	1(Ref)		1(Ref)		1(Ref)		1(Ref)		1(Ref)	
T3	1.34 (1.15 ~ 1.55)	< 0.001	1.32 (1.13 ~ 1.53)	< 0.001	1.25 (1.08 ~ 1.45)	0.003	1.28 (1.1 ~ 1.49)	0.002	1.2 (1.03 ~ 1.4)	0.019

HR, Hazard ratios; GPR, glucose potassium ratio; MAP, mean arterial pressure; RR, respiratory rate; SpO₂, oxygen saturation; Hb, hemoglobin; RBC, red blood cell count; PLT, platelet count; APTT, activated partial thromboplastin time; BUN, blood urea nitrogen; SCr, serum creatinine; Na, sodium; HF, heart failure; PVD, peripheral vascular disease; CVD, cardiovascular disease; DM, diabetes mellitus; GCS, Glasgow Coma Scale; 28d-ACM, 28-day all-cause mortality; 90d-ACM, 90-day all-cause mortality.

Notes: Modle I: Adjust Age and Sex; Modle II: Adjust Modle I + MBP, RR, SpO2, GCS; Modle III: Adjust Modle II + HF, PVD, CVD, Renal disease, Hepatic disorders, DM; Modle IV: Adjust Modle III + Hb, RBC, PLT, APTT, Na, BUN, and SCr.

### The non-linear relationship

**Fig. 2:**
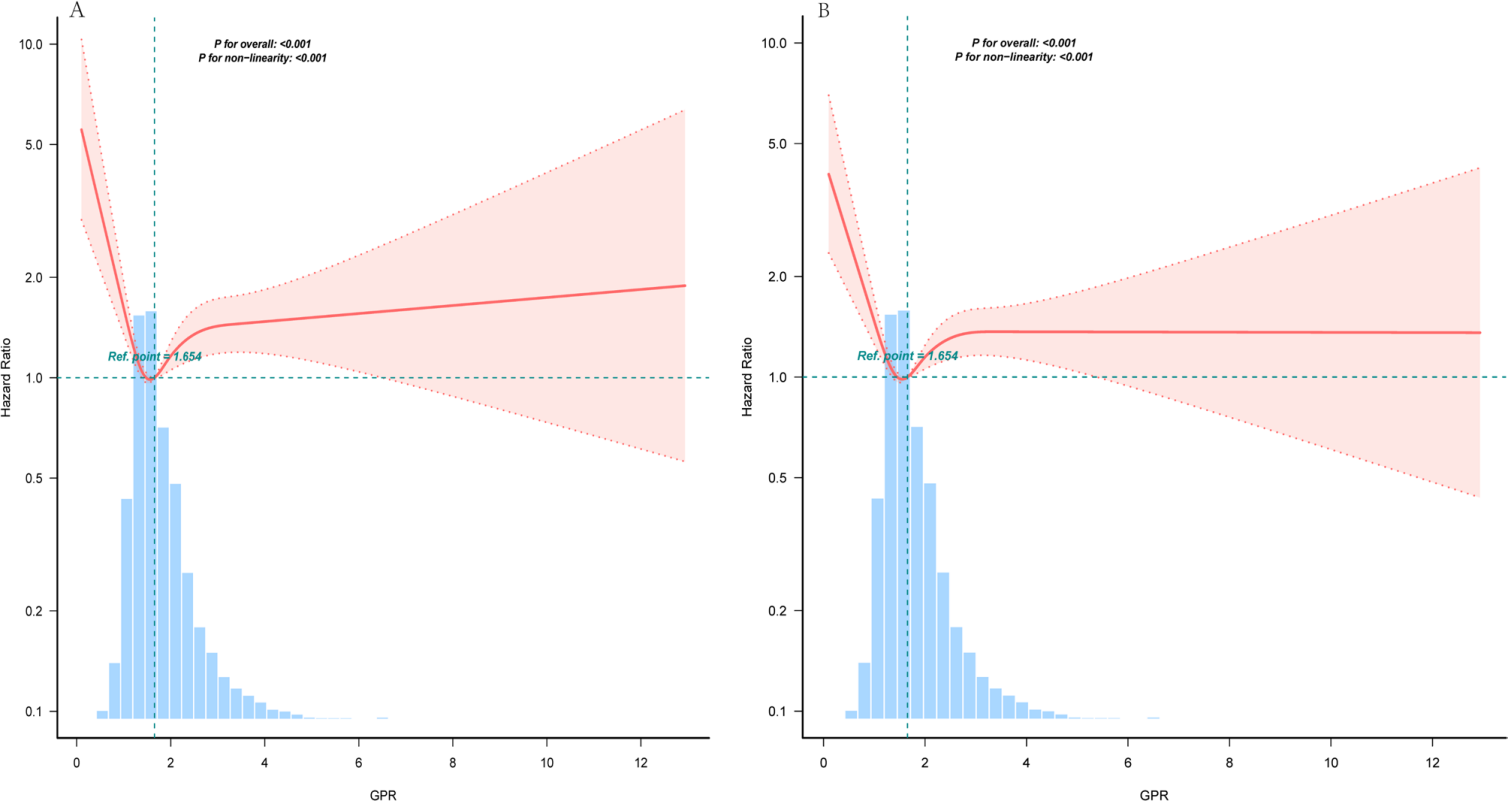
The relationship between GPR level and ACM. Notes: Fig. 2.A for 28-day all-cause mortality, and B for 90-day all-cause mortality. Adjusted for all factors of Model IV (age, sex, MAP, RR, SpO2, GCS, HF, PVD, CVD, Renal disease, Hepatic disorders, DM, Hb, RBC, PLT, APTT, Na, BUN, and SCr). Abbreviations: GPR, glucose potassium ratio; MAP, mean arterial pressure; RR, respiratory rate; SpO₂, oxygen saturation; Hb, hemoglobin; RBC, red blood cell count; PLT, platelet count; APTT, activated partial thromboplastin time; BUN, blood urea nitrogen; SCr, serum creatinine; Na, sodium; HF, heart failure; PVD, peripheral vascular disease; CVD, cardiovascular disease; DM, diabetes mellitus; GCS, Glasgow Coma Scale; ACM, all-cause mortality.

Using multivariate Cox regression analysis and smooth curve fitting, we identified a nonlinear relationship between the GPR levels and ACM (Fig. 2). We applied a piecewise multivariate Cox regression model with two distinct slopes to characterize this association better. The likelihood ratio test yielded a P-value of less than 0.001 (Table 3), thus supporting the use of a two-part model to fit the relationship between GPR levels and ACM in patients with TE. An inflection point is detected at approximately 1.65. For 28-day ACM, the HR on the left side of the inflection point was 0.472 (95% CI: 0.306–0.728, *P* < 0.001), while on the right side, the HR was 1.127 (95% CI: 1.032–1.229, *P* = 0.0075). For 90-day ACM, the HR on the left and right sides of the inflection point were 0.553 (95% CI: 0.381–0.803, *P* = 0.0019) and 1.103 (95% CI: 1.017–1.197, *P* = 0.0179), respectively.

**Table 3:** Threshold effect analysis of GPR and mortality of toxic encephalopathy.

	Adjusted HR (95%CI) ^*^	*P*-value	Adjusted HR (95%CI) ^**^	*P*-value
GPR < 1.65	0.472 (0.306,0.728)	< 0.001	0.553 (0.381,0.803)	0.0019
GPR ≥ 1.65	1.127 (1.032,1.229)	0.0075	1.103 (1.017,1.197)	0.0179
Likelihood Ratio test	-	< 0.001	-	< 0.001

GPR, glucose-potassium ratio; MAP, mean arterial pressure; RR, respiratory rate; SpO₂, oxygen saturation; Hb, hemoglobin; RBC, red blood cell count; PLT, platelet count; APTT, activated partial thromboplastin time; BUN, blood urea nitrogen; SCr, serum creatinine; Na, sodium; HF, heart failure; PVD, peripheral vascular disease; CVD, cardiovascular disease; DM, diabetes mellitus; GCS, Glasgow Coma Scale; HR, Hazard ratios; CI, confidence intervals.

Notes: Adjusted for all factors of Model IV (age, sex, MAP, RR, SpO2, GCS, HF, PVD, CVD, Renal disease, Hepatic disorders, DM, Hb, RBC, PLT, APTT, Na, BUN, and SCr). ^*^28-day all-cause mortality; ^**^90-day all-cause mortality.

### Kaplan–meier survival curve

**Fig. 3:**
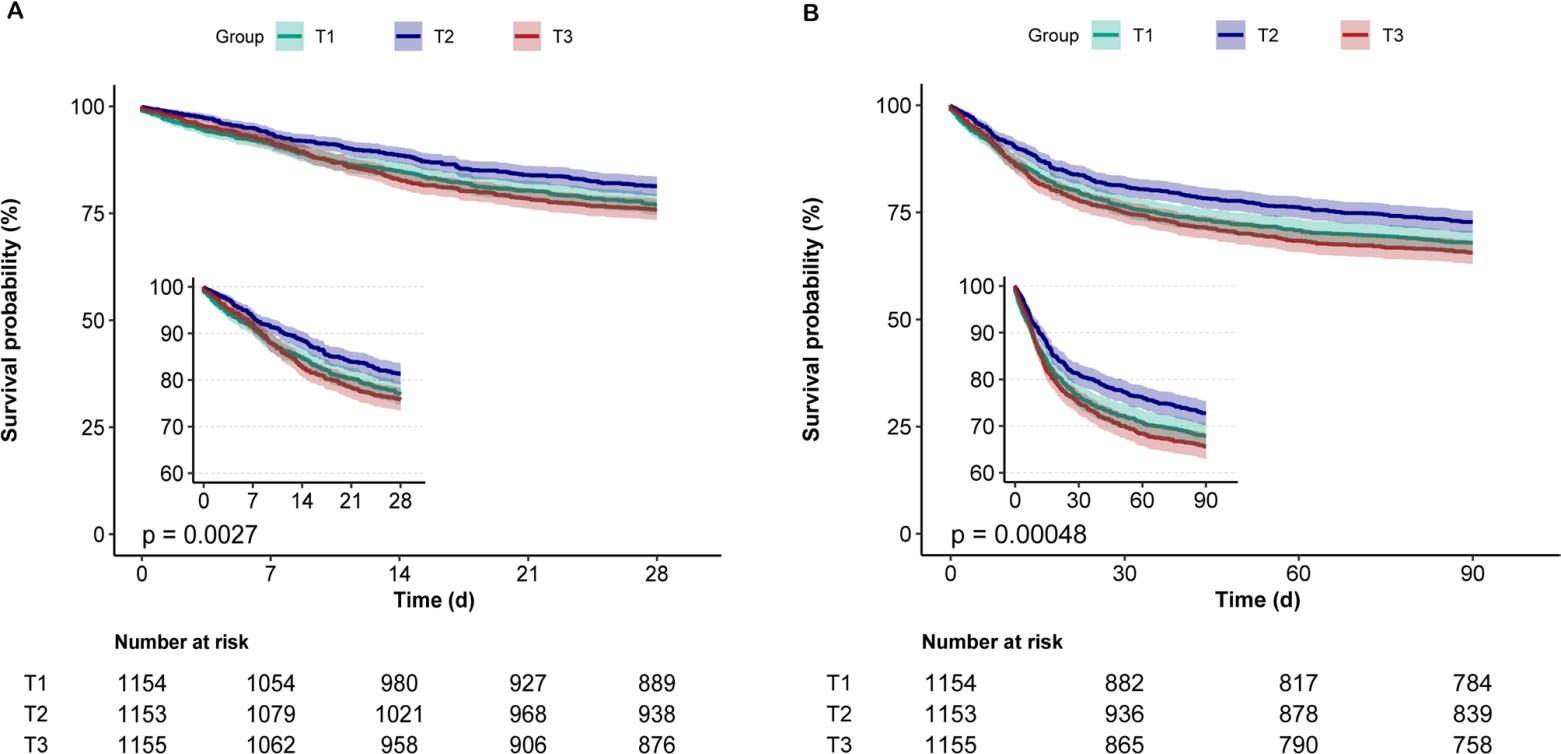
Kaplan–Meier curves indicate the association between the GPR and ACM of TE. The curved line and shaded areas depict the estimated values and their corresponding 95% confidence intervals. Notes: Fig. 3.A for 28-day all-cause mortality, and B for 90-day all-cause mortality. Abbreviations: T1: GPR (0.103–1.442), T2: GPR (1.444–1.966), T3: GPR (1.967–12.937). GPR, glucose potassium ratio; ACM, all-cause mortality; TE, Toxic Encephalopathy; d, day; 28d-ACM, 28-day all-cause mortality; 90d-ACM, 90-day all-cause mortality.

Figure 3.A presents Kaplan-Meier survival curves for GPR levels (T1, T2, and T3) over a 28-day period following hospital admission. Survival probability differed significantly among the groups (*P* = 0.0027), with T3 demonstrating a steeper decline in early survival rates than T1 and T2. The 90-day cumulative mortality rates (Fig. 3. B), similar to that observed at 28 d (*P* = 0.00048).

### Subgroup analysis

**Fig. 4:**
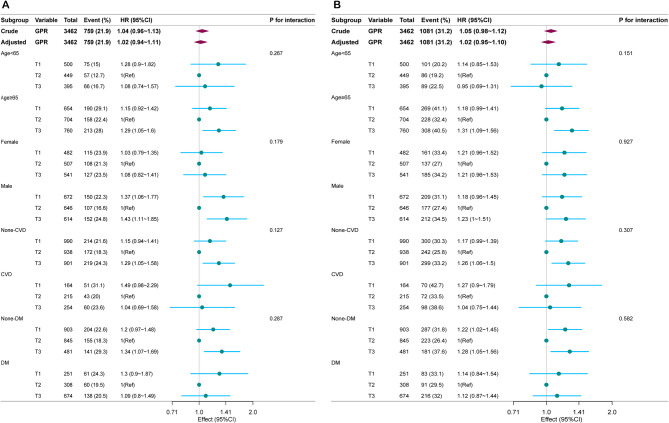
Association between GPR and ACM according to baseline characteristics. Each stratification was adjusted for all factors of Model IV. Notes: Fig. 4.A for 28-day all-cause mortality, and B for 90-day all-cause mortality. Abbreviations: GPR, glucose potassium ratio; CVD, cardiovascular disease; DM, diabetes mellitus; ACM, all-cause mortality; HR, Hazard ratios; CI, confidence intervals.

Subgroup analyses were conducted across several categories, including age, sex, CVD, and DM to evaluate potential effect modifications in the relationship between GPR and ACM. Although there appear to be differences in the HR values of GPR and ACM between the “Age < 65” and “Age ≥ 65” groups, the P-values for interaction (0.267 for age stratification in Figure A and 0.151 in Figure B) indicate that these differences are not statistically significant. Therefore, no significant interactions were observed in any subgroup (Fig. 4).

## Discussion

In this study, we have evaluated the correlation between the GPR and ACM in critically ill patients with TE from the MIMIC-IV database. Multivariate Cox regression analysis showed that when the GPR was used as a continuous variable, its association with the 28-day and 90-day ACM was not significant. Curve-fitting analysis indicated that the relationship between GPR and both the 28-day and 90-day ACM followed a U-shaped pattern with an inflection point at approximately 1.65. Two separate linear regression analyses were performed based on the inflection points. The results showed that GPR was negatively associated with ACM on the left side of the inflection point, whereas a positive correlation was observed on the right side. Additionally, the performed subgroup analyses found no significant interactions between age, sex, CVD, and DM, and the relationship between GPR and ACM. This suggests that GPR’s predictive capacity of the GPR as a prognostic marker remains relatively consistent across the subgroups. Therefore, the GPR may serve as a valuable tool in clinical decision-making and could be considered a prognostic factor for both short- and long-term outcomes following TE.

Our findings are consistent with those of previous studies in regard to the relationship between the GPR and the prognosis of other neurological diseases. The association between the GPR and short- and long-term mortality in patients with acute ischemic stroke (AIS) has been extensively investigated. For example, a Norwegian cohort study including 784 AIS patients showed that GPR was positively correlated with 30-day mortality (OR 2.01, 95% CI: 1.12–3.61, *P* < 0.001), and this relationship remained significant after adjusting for potential confounders^20^. Similarly, in a study evaluating the prognosis of patients with intracerebral hemorrhage (ICH) patients, an elevated GPR was significantly associated with both short-term (30-day) and long-term (1-year) mortality, with a linear relationship^21^. Furthermore, the prognostic value of GPR has also been confirmed in traumatic brain injury (TBI)^8,22^, heart failure^23^, acute type A aortic dissection^24^, acute traumatic spinal cord injury^25^, and DNS after carbon monoxide poisoning^11^. On the left side of the inflection point (GPR < 1.65), we observed a negative correlation between the GPR and ACM. This suggests that lower GPR levels may protect against mortality, possibly due to better metabolic stability and fewer complications. Lower GPR levels may indicate a more balanced metabolic state with neither hyperglycemia nor hypokalemia, which is associated with improved outcomes in critically ill patients. This finding is consistent with those of past studies showing that maintaining optimal glucose and potassium levels is crucial for reducing mortality in ICU settings. For example, previous studies in patients with acute AIS and ICH have reported a protective effect of lower GPR levels on mortality^20,21^.

The potential mechanisms underlying the association between GPR and neurological disease prognosis may be closely related to the physiological roles of glucose and potassium levels. Glucose is the primary source of cellular energy metabolism, and elevated levels may reflect a hyperglycemic response to stress associated with inflammation, oxidative stress, and cellular damage^26^. Potassium plays a key role in maintaining the cell membrane potential and nerve conduction, and hypokalemia may indicate impaired cellular function^27^. In toxic encephalopathy, elevated GPR may reflect the severity of the systemic stress response and metabolic disorders, thereby affecting the repair and recovery of brain tissue.

Moreover, the prognostic value of GPR in different diseases may be influenced by underlying pathophysiological mechanisms. For example, in ischemic and hemorrhagic stroke, the association between GPR and prognosis may be related to stress-induced metabolic disorders and inflammatory responses^20,21^. In methylxanthine poisoning, elevated GPR is closely associated with severe neurological complications (such as seizures and arrhythmias), which may be related to catecholamine release and metabolic disorders caused by methylxanthines^28^. These findings suggest that the GPR, as a composite indicator, may better reflect the overall metabolic state of patients and their prognostic risks than glucose or potassium alone.

GPR is a readily available biomarker with potential clinical applications. However, the specific mechanisms underlying to require further exploration. Future research could focus on the direct association between GPR and cerebral tissue pathophysiological changes, such as verifying its role in neuronal injury and repair using animal models and cell experiments^29^. Additionally, combining genetic polymorphism studies^30,31^ to explore the relationship between GPR and individual susceptibility may provide new insights for precision medicine in toxic encephalopathy.

The strength of our study lies in its scale, as it is the largest retrospective cohort study to date that has investigated the association between GPR and all-cause mortality in patients with TE. However, this study had several limitations. First, as this was a retrospective cohort study, it may have been affected by selection bias and unadjusted confounding factors. Second, only a single numerical ratio of the GPR was used, and its dynamic changes were not monitored, which may have affected the interpretation of the results. Third, the relatively small sample size and single-center design limit the generalizability of the study findings. Future studies should employ multicenter prospective designs to dynamically monitor GPR levels and further validate their prognostic value in different subtypes of toxic encephalopathy.

## Conclusion

In summary, our study suggests that GPR is closely associated with the prognosis of patients with toxic encephalopathy, showing a U-shaped relationship, particularly when the GPR levels are elevated. Future studies should therefore further validate the potential of the GPR as a prognostic marker and explore its clinical applications.

## Electronic supplementary material

Below is the link to the electronic supplementary material.

Supplementary Material 1

## Data Availability

The datasets used and/or analysed during the current study are available from the corresponding author on reasonable request.
