# Supplementary material for: U shaped relationship between serum glucose potassium ratio and mortality in critically ill patients with toxic encephalopathy
Source: Sci Rep. 2025 Jul 23;15:26795. doi: 10.1038/s41598-025-12496-4 (PMC12287516; doi:10.1038/s41598-025-12496-4)
Supplement: Supplementary file 1 — Supplementary Material 1 [file 41598_2025_12496_MOESM1_ESM.docx]

Table S1. The variance inflation factor (VIF) of variables

| Variables | VIF |
| --- | --- |
| Age (year) | 1.644 |
| Male (n, %) | 1.071 |
| MAP (mmHg) | 1.231 |
| RR (breath/min) | 1.339 |
| SpO2 (%) | 1.17 |
| Hb (g/dL) | 4.799 |
| RBC (10^9^/L) | 4.791 |
| PLT (10^9^/L) | 1.271 |
| APTT (sec) | 1.034 |
| BUN (mg/dL) | 2.017 |
| SCr (mg/dL) | 2.146 |
| HF (n, %) | 1.158 |
| PVD (n, %) | 1.04 |
| CVD (n, %) | 1.055 |
| Renal disease (n, %) | 1.387 |
| Hepatic disorders (n, %) | 1.293 |
| DM (n, %) | 1.239 |
| GCS | 1.486 |

Abbreviations: MAP, mean arterial pressure; RR, respiratory rate; SpO₂, oxygen saturation; Hb, hemoglobin; RBC, red blood cell count; PLT, platelet count; APTT, activated partial thromboplastin time; BUN, blood urea nitrogen; SCr, serum creatinine; HF, heart failure; PVD, peripheral vascular disease; CVD, cardiovascular disease; DM, diabetes mellitus; GCS, Glasgow Coma Scale.

**Table S2: Missing Status of Covariates.**

| Variable | Miss.freq | Miss.percentage% |
| --- | --- | --- |
| Na | 1 | 0.03 |
| SCr | 2 | 0.06 |
| GCS | 3 | 0.09 |
| Heart rate | 3 | 0.09 |
| MAP | 3 | 0.09 |
| SOFA | 3 | 0.09 |
| RR | 6 | 0.17 |
| SpO2 | 6 | 0.17 |
| RBC | 10 | 0.29 |
| Hb | 11 | 0.32 |
| PLT | 11 | 0.32 |
| WBC | 12 | 0.35 |
| APTT | 405 | 11.70 |

Abbreviations: Na, sodium; SCr, serum creatinine; GCS, Glasgow Coma Scale; MAP, mean arterial pressure; SOFA, Sequential Organ Failure Assessment; RR, respiratory rate; SpO₂, oxygen saturation; RBC, red blood cell count; Hb, hemoglobin; PLT, platelet count; WBC, white blood cell count; APTT, activated partial thromboplastin time.
